# ADAPTING A VIRTUAL MEDICAL PLANNING PROGRAM FOR CARE PARTNERS OF PERSONS WITH COGNITIVE IMPAIRMENT

**DOI:** 10.1093/geroni/igae098.1302

**Published:** 2024-12-31

**Authors:** Jasmine Santoyo-Olsson, Deborah Barnes, Brookelle Li, Angelica Martinez, Rebecca Sudore

**Affiliations:** University of California San Francisco, San Francisco, California, United States; University of California San Francisco, San Francisco, California, United States; University of California San Francisco, San Francisco, California, United States; University of California San Francisco, San Francisco, California, United States; University of California San Francisco, San Francisco, California, United States

## Abstract

Care partners want to help persons with cognitive impairment (PCI) engage in advance care planning (ACP) before they lose decision-making capacity, but report needing more support and access to virtual information. We created the video-based PREPARE for THEIR Care website to support care partners in helping others with their medical planning. However, PREPARE for THEIR Care has not yet been adapted for online, facilitated, synchronous classes to allow participants to ask facilitators ACP-related questions. We conducted virtual semi-structured interviews with experienced virtual group facilitators (n=4) and care partners of PCI (n=10) recruited from a research study of online exercise group classes for PCI and care partners. We used the Capability, Opportunity, Motivation, Behavior (COM-B) framework to explore barriers, enablers, and implementation recommendations and to guide qualitative thematic analysis. All facilitators were women. Care partner mean age was 62.3 (SD=13.8) years and 70% were women. Barriers included ACP complexity; virtual facilitation complexity; complicated dyad relationships; technology limitations; pessimism; fear of death; and lack of readiness. Enablers included ACP, technology, and teaching experience; participating from home; peer support; normalizing and modeling ACP; and belief that ACP is impactful. Implementation recommendations included providing staff trainings/scripts; care partner technology support before and during sessions; easy-to-use written materials; interactive group activities; and facilitating PREPARE for THEIR Care over multiple short (30-60 minute) sessions with > 50% of the session for discussion. Future ACP interventions should include training for facilitators, easy-to-use ACP materials, technology support, and modelling of ACP in virtual sessions.

